# Supplementary material for: Efficient Hole Transfer from a Twisted Perylenediimide Acceptor to a Conjugated Polymer in Organic Bulk-Heterojunction Solar Cells
Source: Materials (Basel). 2023 Jan 12;16(2):737. doi: 10.3390/ma16020737 (PMC9866189; doi:10.3390/ma16020737)
Supplement: Supplementary file 1 [file materials-16-00737-s001.zip › materials-2143108-supplementary.pdf]

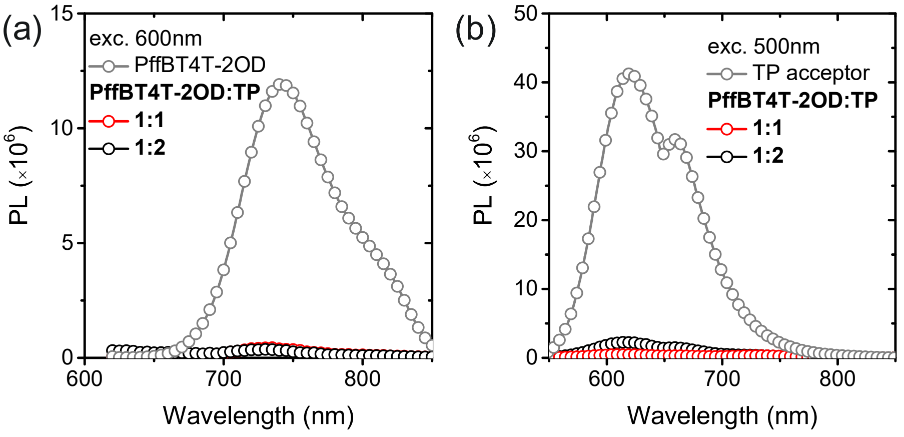

Figure S1. Steady state PL spectra of films (a) excited at 600 nm and (b) excited at 500 nm for pristine PffBT4T-2OD, pristine TP and PffBT4T-2OD:TP blends.

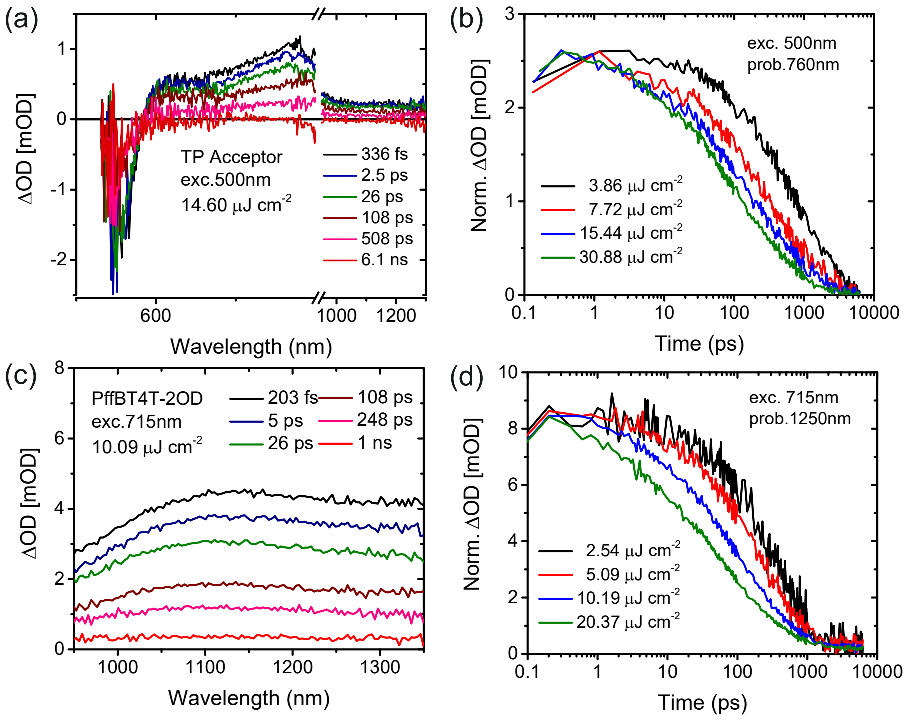

Figure S2. (a) fs-Transient absorption spectra and (b) dynamics probed at 760 nm of neat film of TP excited at 500 nm, and (b) fs-Transient absorption spectra and (d) dynamics probed at 1250 nm of neat film of PffBT4T-2OD excited at 715 nm.

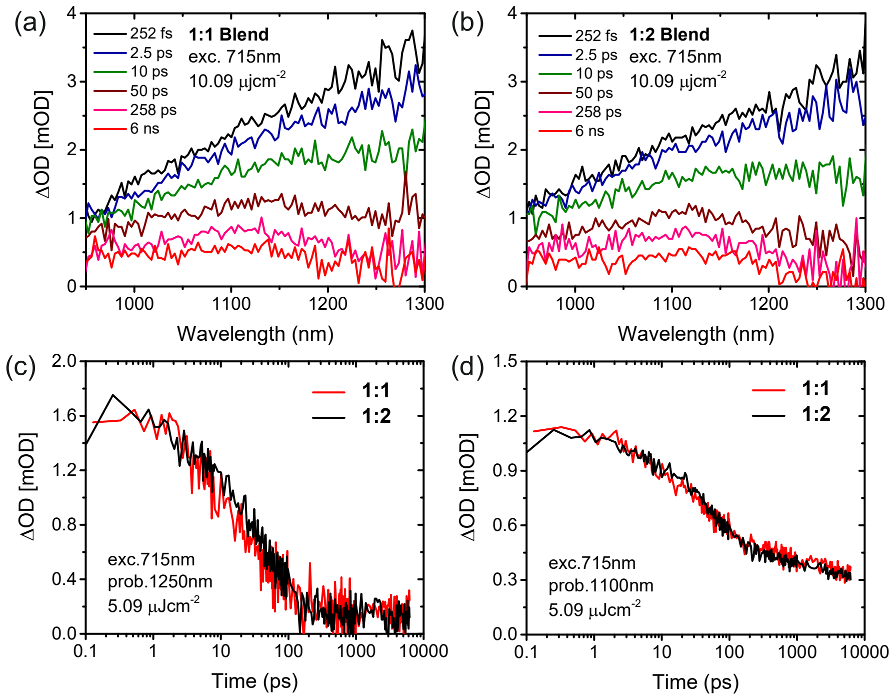

Figure S3. fs-Transient absorption spectra of blends of PffBT4T-2OD:TP of (a) 1:1 and (b) 1:2 weight ratio excited at 715 nm and the dynamics at selected probe of blends of PffBT4T-2OD:TP of 1:1 and 1:2 weight ratio excited at 715 nm: (c) 1250 nm and (d) 1100 nm.

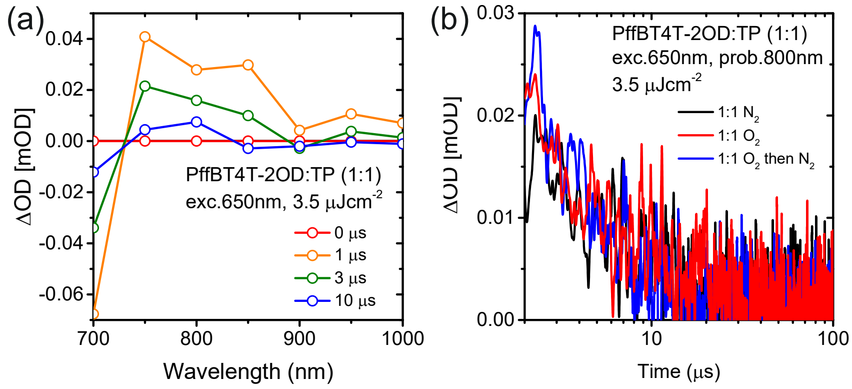

Figure S4. (a) μs-Transient absorption spectra of a PffBT4T-2OD:TP (1:1) blend film, pumped at 650 nm with fluence of 3.5 μJ cm^{-2} and probed at 0-10 μs, and (b) the compares decay dynamics of polaron absorption of PffBT4T-2OD:TP blend at 800 nm.
